# MR-Augmented Cardiopulmonary Exercise Testing- a proof of concept in Sickle Cell Disease (SCD)

**DOI:** 10.1186/1532-429X-18-S1-O69

**Published:** 2016-01-27

**Authors:** Emmanuel O Ako, Nathaniel J Barber, Grzegorz T Kowalik, Jennifer A Steeden, John Porter, John Malcolm Walker, Vivek Muthurangu

**Affiliations:** 1 grid.83440.3b0000000121901201The Hatter Cardiovascular Institute, University College London, London, UK; 2 Great Ormond Street Hospital for Children, Institute of Cardiovascular Science, London, UK

## Background

Exercise intolerance is a common feature of many non-cardiac and non-respiratory diseases. The causes are often multifactorial and include secondary cardiac-respiratory dysfunction, as well as skeletal muscle abnormalities. Unfortunately, it is difficult to determine the exact cause using conventional cardiopulmonary exercise testing (CPET). Therefore, we have developed MR augmented CPET that allows simultaneous evaluation of cardiac output and tissue oxygen extraction in addition to conventional CPET measures. To demonstrate the utility of this technique we performed MR-CPET on patients with sickle cell disease (SCD). The aim of this study was to demonstrate that MR-CPET could be used to define the physiological factors associated with their poorly understood exercise intolerance.

## Methods

14 patients with homozygous sickle cell disease (age: 30-41) and 14 healthy volunteers (age: 25-37) underwent MR-CPET. Exercise was performed on MR-compatible ergometer (Lode, Groningen, The Netherlands) and minute ventilation (VE), oxygen consumption (VO_2)_, and carbon dioxide production (VCO_2)_ were assessed using a commercial respiratory gas analyser (Ultima, MedGraphics, St. Paul, USA) with modified sampling tube that was MR compatible. Aortic flow was simultaneously continuously measured using a previously validated real-time UNFOLD-SENSE spiral PCMR sequence. MR data was used to derive cardiac output (CO), heart rate (HR) and stroke volume (SV) curves during exercise. Arteriovenous oxygen content gradient (AVO_2_) curves (a measure of tissue oxygen extraction) were calculated by dividing the VO_2_ and CO curves.

## Results

**Figure 1:**
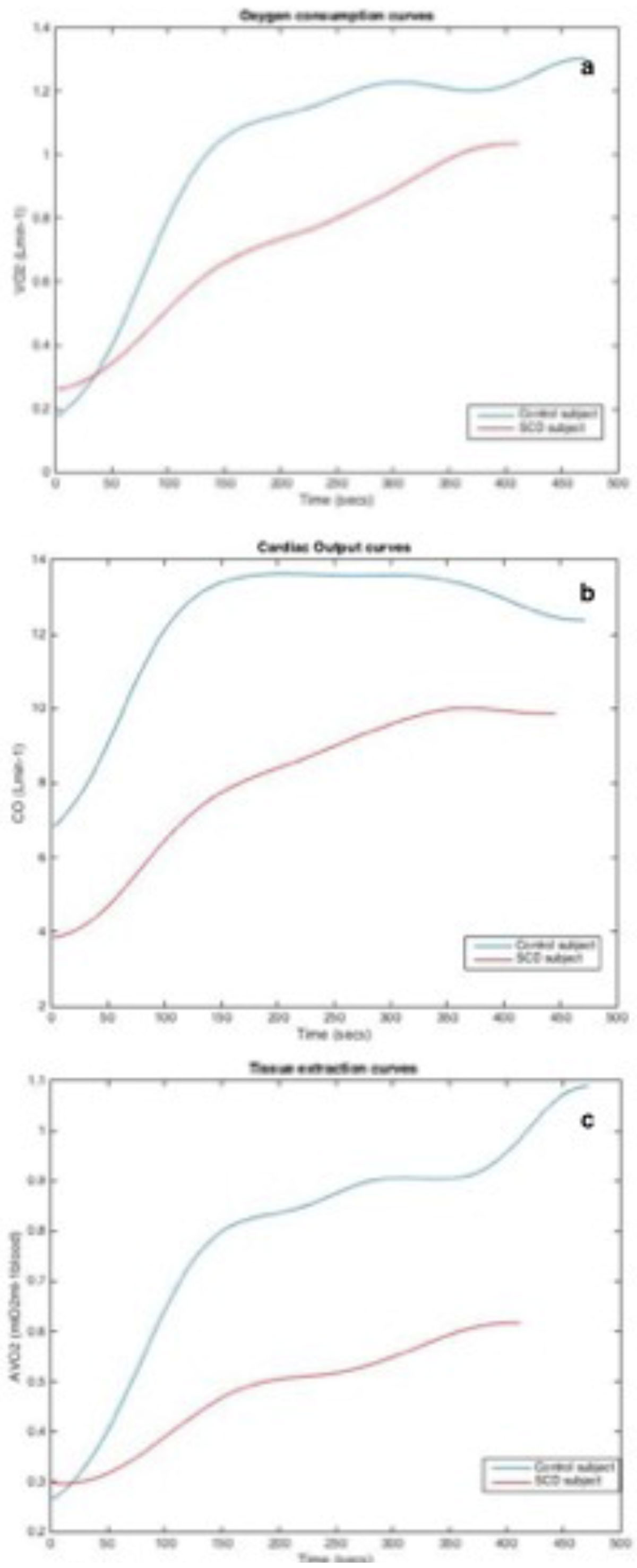
**(a) Oxygen consumption (b) Cardiac Output and (c) Tissue extraction during exercise**.

All participants completed exercise with no adverse outcome including the sickle group. MR-CPET measures at rest and exercise are shown in table 1. The main finding was that peak VO_2_ was significantly lower in patients (fig. 1a). This was partly driven by a reduced CO response (fig. 1b) in SCD patients, due to a lower peak heart rate. However, linear regression analysis demonstrated that reduced AVO_2_ response (fig. 1c) was the main driver of reduced peak VO2 (p-0.018) in patients.

**Table 1:** Resting and peak values during MR-CPET

Variable	Normal Mean (range)	Disease Mean (range)	P-value
Resting VO2, *Lmin*^*-1*^	0.210 (0.17- 0.25)	0.231 (0.19-0.27)	0.38
Peak VO2, *Lmin*^*-1*^	1.1 (0.9- 1.3)	0.7 (0.56- 0.76)	0 < 0.001**
Resting cardiac output, *Lmin*^*-1*^	6.7 (5.9-7.5)	8.1 (7.1-9.2)	< 0.05*
Peak cardiac output, *Lmin*^*-1*^	13 (12-14)	12 (11-13)	< 0.05*
Resting stroke volume, *mlbeat*^*-1*^	98 (84-111)	109 (98-119)	0.17
Peak Stroke volume, *mlbeat*^*-1*^	101 (81-120)	116 (105- 27)	0.16
Resting heart rate, *bpm*	66 (56-77)	77 (72-81)	0.067
Max heart rate, *bpm*	138 (124-152)	120 (111-129)	< 0.05*
Resting tissue extraction, *mlO2ml-1blood*	0.31 (0.26-0.35)	0.28 (0.24-0.32)	0.34
Peak tissue extraction, *mlO2ml-1blood*	0.90 (0.79-1.02)	0.51 (0.43- 0.59)	< 0.001**

## Conclusions

Using MR-CPET we have been able to show for the first time that exercise intolerance in SCD is due to reduced skeletal muscle oxygen extraction. This may be due to vascular network rarefaction, muscle fibrosis, or reduced mitochondrial function; all of which have been demonstrated in histology specimens in SCD. Without simultaneous CO measures it is would not have been possible to demonstrate the importance of reduced tissue extraction. This demonstrates the power of MR-CPET and we believe this technique could aid in better understanding of exercise intolerance and possibly better therapeutic interventions.

